# Stoichiometry of irreversible ligand binding to a one-dimensional lattice

**DOI:** 10.1038/s41598-020-77896-0

**Published:** 2020-12-04

**Authors:** Philipp O. Tsvetkov

**Affiliations:** CNRS, UMR 7051, INP, Inst Neurophysiopathol, Fac Médecine, Aix Marseille Univ, Marseille, France

**Keywords:** Biophysics, Computational biology and bioinformatics

## Abstract

In this paper we investigate the problem of irreversible binding of a ligand that covers several identical binding sites on a macromolecule with a one-dimensional lattice. Due to steric constraints, irreversible binding or binding with slow kinetics results in partial saturation of the binding sites thus impacting the stoichiometry of the interaction. Here we present a recursive formula to calculate the exact fraction of the occupied binding sites for a ligand and macromolecule of arbitrary lengths. We also provide an analytical result for the exact fraction of the occupied sites in case of an infinitely long lattice. We conclude with a simplified empirical formula for the exact fraction of the occupied sites in case of an infinitely long lattice.

## Introduction

One-dimensional lattice-like models of interaction are used to study the ligand binding to regular macromolecules containing long repetitive sequences of binding sites (binding lattice). Usually, such models are used to study DNA/RNA-ligand interactions^1^, but are also applicable for analyses of polysaccharide^2^ and supramolecular polymers such as microfilaments and microtubules^3^. The equilibrium ligand binding to a one-dimensional lattice is usually described by the McGhee–von-Hippel equation^4^. Unfortunately, due to the slow kinetics of the interaction, a transient (pseudo-equilibrium) state could be misinterpreted as a true equilibrium. In extreme case of irreversible ligand binding, this problem can be described in terms of the so-called car parking problem. The likelihood of the problem occurring also increases with the increase in length of the acceptor lattice^5^. In this paper we investigate how the parking problem constraints influence the stoichiometry of ligand–macromolecule interactions in the case of the irreversible binding. We then derive a closed form solution for an extreme case where the length of the lattice increases infinitely.

If a ligand occupies only one site on the lattice and does not influence the other binding sites (non-cooperative binding), its interactions can be described by a simple model of binding with the stoichiometry 1:1. However, if the ligand occupies several neighboring binding sites on the macromolecule, the full saturation cannot be reached. Indeed, ligands binding to available sites at random can lead to formation of areas on the lattice where free sites cannot be occupied by the ligand molecule due to steric constraints (e.g., gap size is smaller than that of the ligand). For example, if the ligand occupies ℓ binding sites after saturating the regions with 1, …, ℓ − 1, the remaining free binding sites will stay unoccupied because they cannot accept ℓ-long ligand. The probability of the lattice becoming fully covered by ligands decreases dramatically with the decrease of the lattice length (A231580 in^8^). This means that the stoichiometry of such interactions (the maximal ratio between bound binding sites and ligand) will always be higher than expected. In this study we do not consider models of the ligand binding to the lattice but determine the stoichiometry of such interactions through the ligand-to-binding-sites molar ratio after the saturation is reached.

## Results

**Figure 1:**
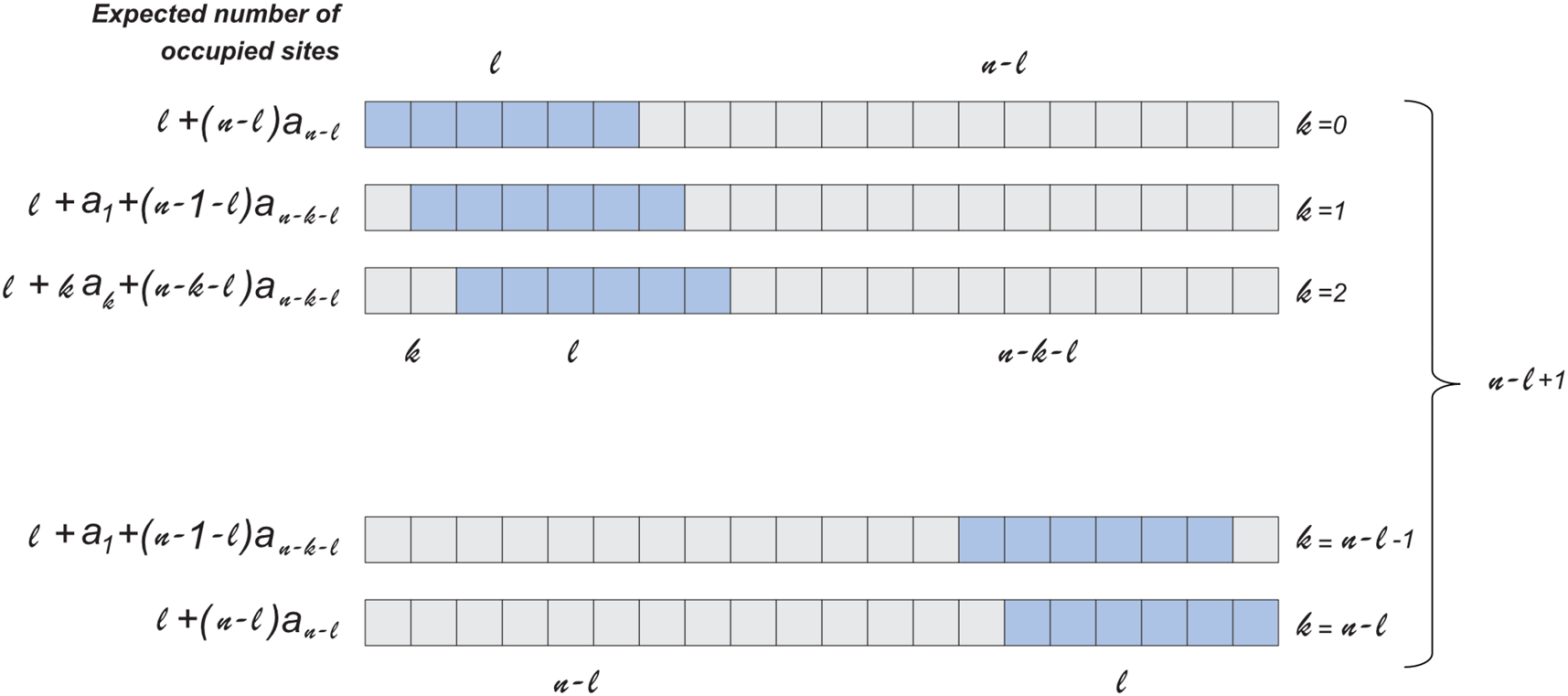
The random filling of n-length lattice by ℓ-length segments. First segment (shown in light blue) has an equal probability to occupy one of the n − ℓ + 1 position shown in figure. The expected number of occupied sites after the complete filling of the lattice for each configuration will be equal to ℓ plus the expected number of occupied sites for lattice on the right and on the left from the first segment.

To solve this problem, we will consider a n-long lattice randomly filled with a ℓ-long ligand. Let $a_{l, n}$ be the expected fraction of sites occupied by the ℓ-long ligand at the n-long lattice. Obviously, if the length of the lattice is less than the length of the ligand then the fraction of occupied sites is equal to zero $(a_{l, n} = 0, for n < l),$ and when the lengths of the lattice and ligand are equal then all sites will be occupied $\left( {a_{l, n} = 1, for n = l} \right)$. For a larger lattice lengths (*n* > *l*), the fraction of the occupied sites (Fig. 1) could be found by modifying the difference equation used by Gordon^6^ for the number of vacant sites, which is in turn a generalization of the simplest case of 2-long ligand^7^:

1 $$ a_{l,n} = \frac{{l + 2\left( {n - l} \right)a_{l,n - l} + \left( {n - 1} \right)\left( {n - l} \right)a_{l,n - 1} }}{{n\left( {n + 1 - l} \right)}}. $$

**Figure 2:**
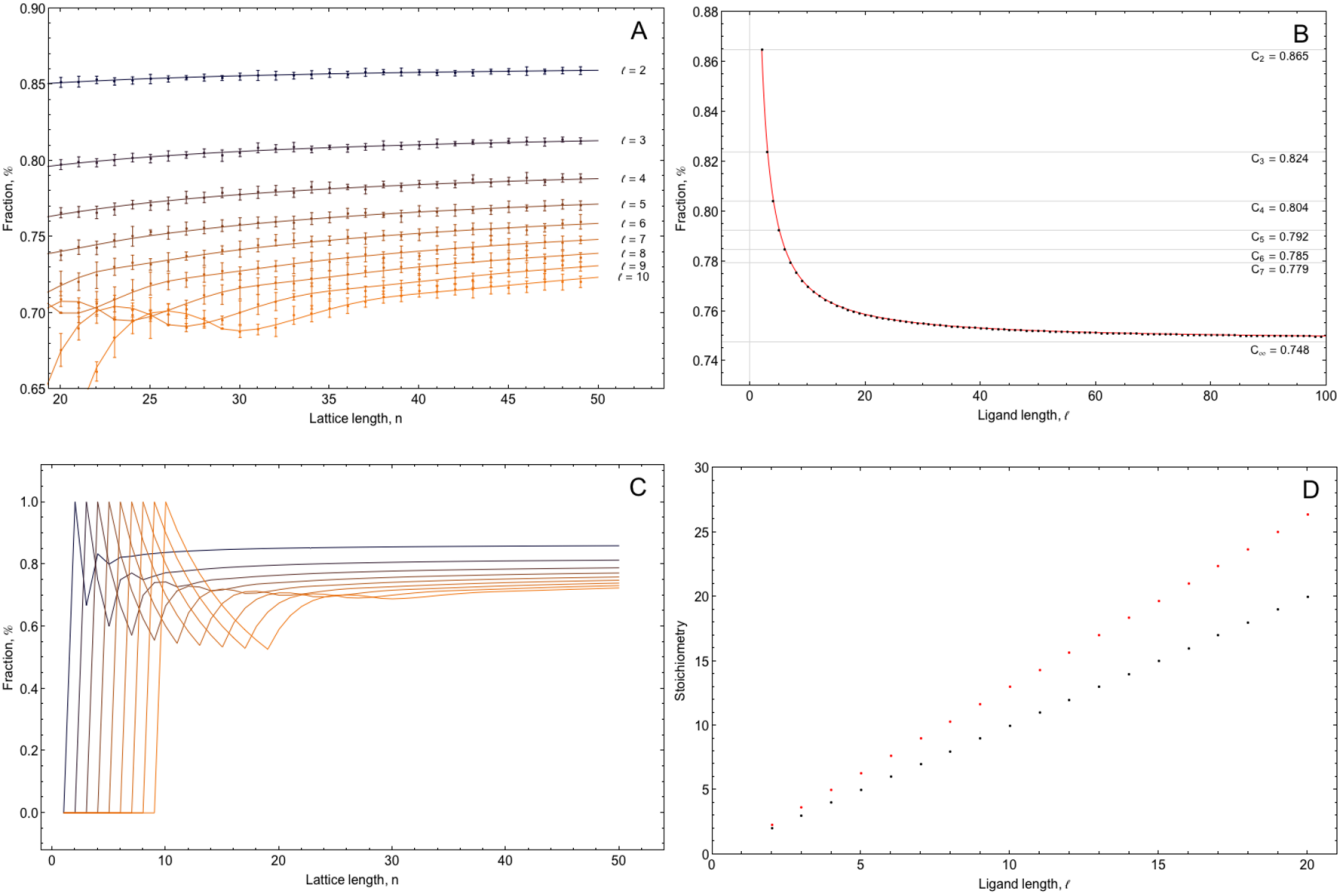
(**A**) Fraction of occupied sites on n-length lattice for different values of ligand length (ℓ). Theoretical curves are shown in solid lines and numerical simulation values (see “Method” section for details) in squares with error bars. (**B**) The fraction of occupied sites on infinite lattice as a function of ligand length is shown in black squares and best fit with Eq. (8) as red curve. (**C**) Fraction of occupied sites of n-length lattice for different values of ligand length (ℓ) from 2 to 10. (**D**) Stoichiometry of ℓ-length ligand binding on infinite lattice in case of equilibrium (black dots) and irreversible (red dots) intercaction.

The dependences between the fraction of the occupied sites and the length of the lattice (n) is presented in Fig. 2A,C for different values of ligand length (ℓ). Theoretical curves derived from Eq. (1) are shown by solid lines and simulation values (when a n-long lattice is randomly filled with ℓ-long ligand 1000 times) are shown by squares with error bars attached.

Together with known $a_{l, n}$ for $n < l$ this relation could be used to generate recurrence sequence A^ℓ^ = {$a_{l,0}$, $a_{l,1}$, $a_{l,2}$, …} to find the fraction of sites occupied by a ℓ-long ligand binding to the lattice of any length. Let's solve this recurrence using generating functions approach. For this, we multiply the Eq. (1) by *x*^*n*^ and sum for all n from ℓ to ∞:

2 $$ \mathop \sum \limits_{n = l}^{\infty } n\left( {n + 1 - l} \right)a_{l,n} x_{{}}^{n} = \mathop \sum \limits_{n = l}^{\infty } lx_{{}}^{n} + \mathop \sum \limits_{n = l}^{\infty } 2\left( {n - l} \right)a_{l,n - l} x_{{}}^{n} + \mathop \sum \limits_{n = l}^{\infty } \left( {n - 1} \right)\left( {n - l} \right)a_{l,n - 1} x_{{}}^{n} $$

If we define *A*_*l*_*(x)* as $\sum\nolimits_{n = 0}^{\infty } {a_{l,n} } x^{n}$ and take into account that $A_{l}^{\prime } \left( x \right) = \sum\nolimits_{n = 1}^{\infty } n a_{l,n} x^{n - 1}$ and that $A_{l}^{\prime \prime } \left( x \right) = \sum\nolimits_{n = 2}^{\infty } {n\left( {n - 1} \right)a_{l,n} x^{n - 2} }$ we can rewrite Eq. (2) as differential equation:

3 $$ \left( {x^{2} A_{l}^{\prime \prime } \left( x \right) - \left( {2 - l} \right)xA_{l}^{\prime } \left( x \right)} \right)\left( {x - 1} \right) + 2x^{l + 1} A_{l}^{\prime } \left( x \right) + \frac{{lx^{l} }}{1 - x} = 0 $$

It is easy to demonstrate that the following function is the solution of the obtained differential Eq. (3):

4 $$ C_{1} + \int_{1}^{x} {\left( {\frac{{e^{{ - 2\mu \left( {t,l} \right)}} t^{l - 2} }}{{\left( {t - 1} \right)^{2} }}\left( {C_{2} + \int_{1}^{t} {le^{{2\mu \left( {k,l} \right)}} dk} } \right)} \right)dt,} $$

where $\mu \left( {x,l} \right) = \sum\nolimits_{n = 1}^{l - 1} {\frac{{x^{n} }}{n}}$ and C_1_ and C_2_ are constants.

Taking into account that $\frac{{A_{l}^{\left( n \right)} \left( 0 \right)}}{n!} = a_{l,n}$ and that $a_{l,n} = 0$ for *n* < ℓ, we can find both C_1_ and C_2_ and finally find *A*_*l*_(*x*):

5 $$ A_{l} \left( x \right) = \int_{0}^{x} {\left( {\frac{{e^{{ - 2\mu \left( {t,l} \right)}} t^{l - 2} }}{{\left( {t - 1} \right)^{2} }}\int_{0}^{t} l e^{{2\mu \left( {k,l} \right)}} dk} \right)} dt. $$

This analytical solution allows to calculate the fraction of the occupied sites by the ℓ-long ligand on the n-long lattice ($a_{l,n}$) for any ℓ and n without using a recurrence sequence. For example, for 2-long ligand the fraction of occupied sites is equal to:

6 $$ a_{2,n} = \frac{{ - \left( { - 2} \right)^{1 + n} e^{2} + \Gamma \left( {3 + n, - 2} \right)}}{{n e^{2} \Gamma \left( {2 + n} \right)}}, $$

where $\Gamma \left( n \right)$ and $\Gamma \left( {n,z} \right)$ are the Euler gamma function and the incomplete gamma function respectively. It also allows to find analytical solution for an extreme case when the lattice length approaches infinity:

7 $$ \begin{aligned} a_{l,\infty } & = le^{{ - 2\mu \left( {1,l} \right)}} \int_{0}^{1} {e^{{2\mu \left( {t,l} \right)}} } dt,\;\;{\text{or}} \\ a_{l,\infty } & = le^{{ - 2H_{l - 1} }} \int_{0}^{1} {e^{{2\mu \left( {t,l} \right)}} } dt, \\ \end{aligned} $$

where $H_{l}$ is a harmonic number.

## Discussion

The car parking problem could considerably impact the stoichiometry of the irreversible (in some cases also reversible) ligand binding to the lattice of binding sites. This problem was theoretically investigated about 80 years ago for a 2-long ligand in the context of intramolecular reaction between neighbouring subtituentes of vinyl polymers by Paul J. Flory^7^, who found the fraction of the occupied sites on a randomly filled infinite lattice to be equal to 1−e^−2^ ⋍ 0.865 (A219863 in OEIS^8^). Further, in 1963 Gordon and Hillier extended this approach to ligands of any length but could not find an analytical solution for an infinite lattice^6^. In case of infinitely long ligand this problems turns into a continuous parking problem and have been solved by Renyi^9^. In this case the fraction of the occupied space called also the Rényi's parking constant is minimal and equal approximately to 0.748 (A050996 in OEIS^8^). General formula (7) presented here allows to calculate the fraction of sites occupied on an infinite lattice by a fixed-length ligand (Fig. 2B). For example, for a ligand lengths equal to 3 and 4, this fraction is equal to ⋍ 0.824 (A307154 in OEIS^8^) and ⋍ 0.804 (A307184 in OEIS^8^) respectively. For ligand lengths equal to 2 or 3 this fraction can be expressed through closed formulae (1−e^−2^ and $\frac{3\sqrt \pi }{{2e^{4} }}\left( {erfi\left( 2 \right) - erfi\left( 1 \right)} \right)$, respectively), and for larger ligand lengths can be estimated numerically from (7). The dependence of $a_{l,\infty }$ as a function of the ligand length ℓ is presented in Fig. 2B. The Eq. (7) can be also approximated for any ℓ (Fig. 2B) with the following formula:

8 $$ a_{l,\infty } \simeq 0.748 + \frac{0.228}{l} \pm 10^{ - 3} . $$

This approximation is applicable for any ligand length in case of a very long lattice. To calculate the precise stoichiometry of the lattice of any length the recurrence Eq. (1) can be used. The difference in the number of sites occupied by a ℓ-long ligand in case of an equilibrium binding (at saturating concentrations) and irreversible binding can be seen in Fig. 2D. For example, a 5-long ligand can occupy 6.3 binding sites on average. Therefore, finding an unexpectedly high stoichiometry (using isothermal titration calorimetry, for example) could be an indication of an irreversible binding or a state, close to pseudo-equilibrium formula presented here allows to find the correct stoichiometry.

In conclusion, here we present the exact analytical formula to calculate the fraction of occupied sites when an infinite lattice randomly filled with ligands of a fixed length (7). Additionally, we present an empiric expression that allows us to estimate this fraction with high precision.

## Method

Fraction of occupied sites on n-length lattice after random fill with ℓ-length ligand was simulated using Wolfram Mathematica 10.1 software (https://www.wolfram.com/mathematica/) for 19 < n < 50 and 1 < ℓ < 11. Each simulation was run 100 times then average value and standard deviation were calculated for all values n and ℓ.

## References

[1] Latt SA, Sober HA (1967). Protein–nucleic acid interactions. II. Oligopeptide-polyribonucleotide binding studies. Biochemistry.

[2] Olson ST, Halvorson HR, Björk I (1991). Quantitative characterization of the thrombin–heparin interaction. Discrimination between specific and nonspecific binding models. J. Biol. Chem..

[3] Tsvetkov PO, Makarov AA, Malesinski S, Peyrot V, Devred F (2012). New insights into tau-microtubules interaction revealed by isothermal titration calorimetry. Biochimie.

[4] McGhee JD, von Hippel PH (1974). Theoretical aspects of DNA–protein interactions: Co-operative and non-co-operative binding of large ligands to a one-dimensional homogeneous lattice. J. Mol. Biol..

[5] Munro PD, Jackson CM, Winzor DJ (1998). On the need to consider kinetic as well as thermodynamic consequences of the parking problem in quantitative studies of nonspecific binding between proteins and linear polymer chains. Biophys. Chem..

[6] Gordon M, Hillier IH (1963). Statistics of random placement, subject to restrictions, on a linear lattice. J. Chem. Phys..

[7] Flory PJ (1939). Intramolecular reaction between neighboring substituents of vinyl polymers. J. Am. Chem. Soc..

[8] Sloane, N. J. A. *The On-Line Encyclopedia of Integer Sequences* http://oeis.org/.

[9] Rényi A (1958). On a one-dimensional problem concerning random place filling. Magyar Tud Akad Mat Intézeténik Kozleményei.

